# Prediction of the Time Course of Callus Stiffness as a Function of Mechanical Parameters in Experimental Rat Fracture Healing Studies - A Numerical Study

**DOI:** 10.1371/journal.pone.0115695

**Published:** 2014-12-22

**Authors:** Tim Wehner, Malte Steiner, Anita Ignatius, Lutz Claes

**Affiliations:** Institute of Orthopedic Research and Biomechanics, Center of Musculoskeletal Research Ulm, University Hospital Ulm, Ulm, Germany; University of Zurich, Switzerland

## Abstract

Numerous experimental fracture healing studies are performed on rats, in which different experimental, mechanical parameters are applied, thereby prohibiting direct comparison between each other. Numerical fracture healing simulation models are able to predict courses of fracture healing and offer support for pre-planning animal experiments and for post-hoc comparison between outcomes of different *in vivo* studies. The aims of this study are to adapt a pre-existing fracture healing simulation algorithm for sheep and humans to the rat, to corroborate it using the data of numerous different rat experiments, and to provide healing predictions for future rat experiments. First, material properties of different tissue types involved were adjusted by comparing experimentally measured callus stiffness to respective simulated values obtained in three finite element (FE) models. This yielded values for Young’s moduli of cortical bone, woven bone, cartilage, and connective tissue of 15,750 MPa, 1,000 MPa, 5 MPa, and 1 MPa, respectively. Next, thresholds in the underlying mechanoregulatory tissue differentiation rules were calibrated by modifying model parameters so that predicted fracture callus stiffness matched experimental data from a study that used rigid and flexible fixators. This resulted in strain thresholds at higher magnitudes than in models for sheep and humans. The resulting numerical model was then used to simulate numerous fracture healing scenarios from literature, showing a considerable mismatch in only 6 of 21 cases. Based on this corroborated model, a fit curve function was derived which predicts the increase of callus stiffness dependent on bodyweight, fixation stiffness, and fracture gap size. By mathematically predicting the time course of the healing process prior to the animal studies, the data presented in this work provides support for planning new fracture healing experiments in rats. Furthermore, it allows one to transfer and compare new *in vivo* findings to previously performed studies with differing mechanical parameters.

## Introduction

The rat is of particular importance for experimental studies in fracture healing. The outcome of the healing process is often evaluated via determination of the callus stiffness by *ex vivo* mechanical testing at certain healing time points [1], [2], [3], [4], [5]. Mechanical and biological factors influence the manner in which fracture healing progresses [6], [7], [8]. The most important mechanical factor is the interfragmentary movement (IFM) [6]; IFM determines the tissue strains at the fracture site in relation to the fracture gap size and regulates the mechanically induced tissue differentiation [9], [10]. The IFM depends on the fixation stability and the musculoskeletal loads which are primarily dependent on the bodyweight of the animals [11]. Therefore, experimental studies in rats are not directly comparable with each other when different experimental parameters (bodyweight, fixation stiffness, fracture gap size) are used.

Over the last decade, several numerical models for simulating the fracture healing process were developed [12], [13], [14], [15], [16], [17], [18]. Those models, which take the experimental parameters mentioned above into consideration, have the potential to simulate the healing process prior to an *in vivo* investigation helping to refine and reduce the number of animals in experimental studies as well as provide comparability between different studies by appropriate pre-experiment planning. Furthermore, simulations can be used to normalize already published *in vivo* results by simulating the healing process with the specific set of experimental parameters. Thereby, they help decide if there is an interesting biological factor influencing the healing outcome or if the differences of the healing outcome between studies can be explained solely by mechanical parameters that influence tissue strains (fixation stiffness, bodyweight, fracture geometry).

The aims of this study were (a) to adapt an existing numerical algorithm that reasonably simulates fracture healing in sheep [18], [19] and in humans [20], [21] to the conditions found in rats, which includes the determination of callus tissue properties and the calibration of the underlying mechanoregulatory tissue differentiation rules, (b) to simulate *in vivo* studies from literature and evaluate their agreement and (c) to predict the time course of the callus stiffness under several combinations of fixation stability, bodyweight, and fracture geometry. For future fracture healing studies using rats, this should promote the replacement of experimental *in vivo* studies, and allow focus on the influence of biological factors.

## Methods

### Determining the material properties of the callus tissues

**Figure 1:**
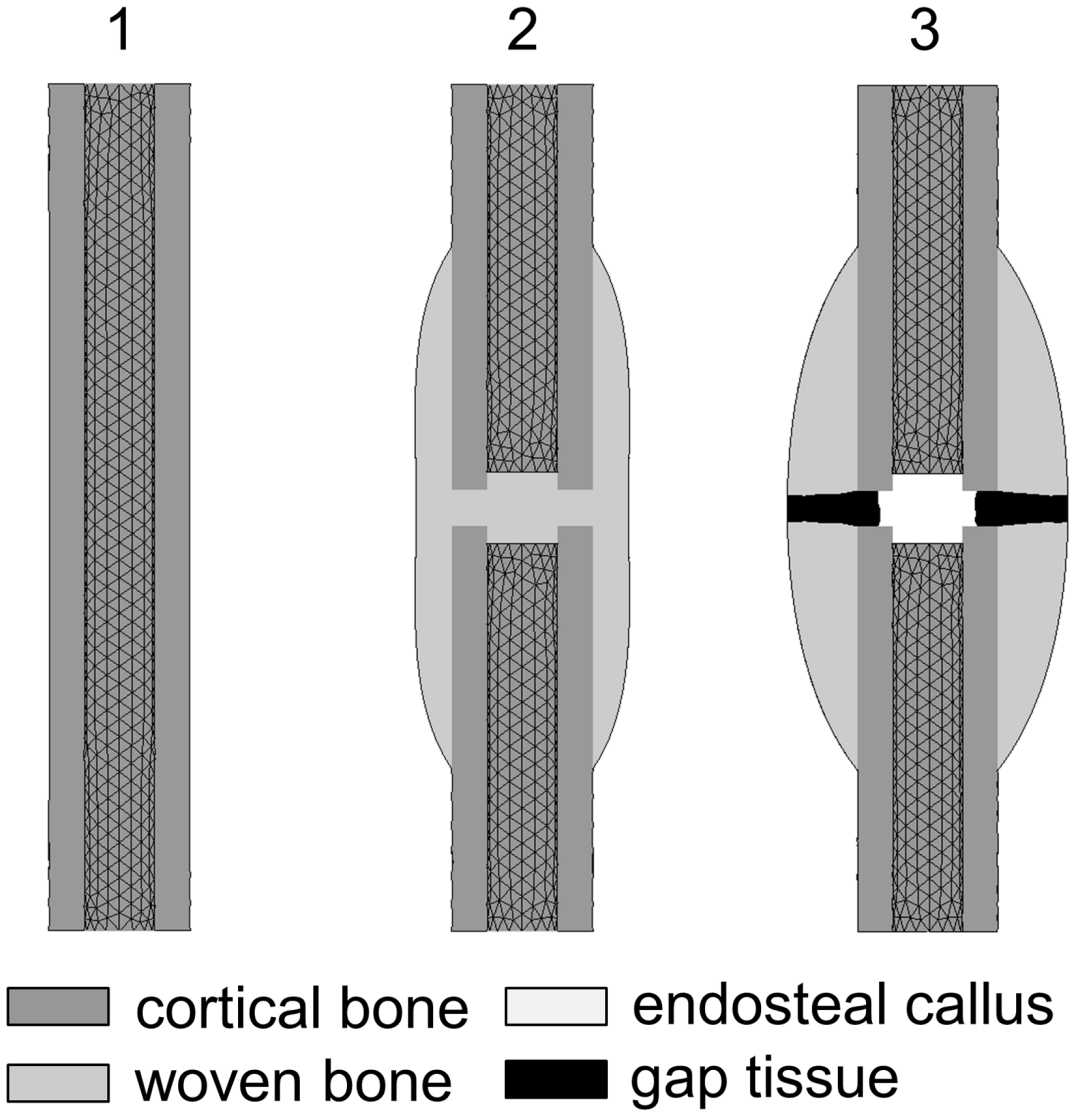
Three finite element models for bending stiffness calculation of three different healing scenarios. Model 1: intact bone cylinder, model 2: fracture callus filled with woven bone and a callus index of 1.5, and model 3: fracture callus with a callus index of 2, where periosteal callus is filled with woven bone up to the fracture line.



To determine the material properties of the different callus tissues involved (*i.e.* connective tissue, cartilage, woven and cortical bone), three FE models of different healing stages were created in ANSYS 14.0 (ANSYS Inc., Canonsburg, PA, USA) to calculate the respective callus bending stiffness. All geometries represented transverse osteotomies at the rat femur in accordance with Fig. 1: (1) intact bone cylinder, (2) callus filled with woven bone according to previous histological findings under rigid fixation after 35 days of healing [2], (3) callus filled with woven bone except within the fracture gap, which was filled with cartilage (periosteal callus) and connective tissue (endosteal callus) according to previous histological findings under flexible fixation after 35 days of healing [2]. Conforming to Checa *et al*. [22], the cortical bones were modeled as hollow cylinders with an inner diameter of 2 mm and an outer diameter of 4 mm. The overall height (*l*) of the models was 24 mm. All structures were meshed with 10-node tetrahedral elements assuming linear-elastic, isotropic material properties. The models were fixed at the bottom (distal end of the distal cortical bone fragment) in all degrees of freedom. To simulate the bending stiffness of the models in a cantilever bending mode, a shear deflection of 1 mm was applied to the top of the models. Therefore, a tent-like structure made of rigid beam elements [21] was used at the top of the models to allow the application of the shear deflection *U*_s_ and the calculation of the reaction force *F*_s_ at one single node. The bending stiffness, *EI*_FE_, was calculated based on the formula for cantilever bending:

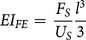
(1)These three FE models were used to successively obtain the material properties by comparing the simulated bending stiffness *EI*_FE_, resulting from various parameter combinations to the respective *ex vivo* bending stiffness *EI*_EX_ measured in rat experiments by Recknagel *et al.* [2] Thus, model (1) was used to obtain Young’s modulus for cortical bone *E*_cort_, which was then assigned to model (2). This was then used to obtain Young’s modulus for woven bone *E*_bone_, which subsequently was assigned to model (3), to obtain Young’s moduli for endosteal callus *E*_endost_, and gap tissue *E*_gap_, as listed in the supporting information (cf. S1 Table).

### Numerical simulation of the fracture healing process

To simulate the fracture healing process, the previously obtained material properties were assigned to another callus FE model with an ellipsoid shaped healing region with an outer diameter of 10 mm, which allowed a maximum callus index [23] (ratio between the outer callus diameter and the periosteal diameter of the cortices) of 2.5. The external fixator and the fixator pins were modeled with 2-node beam elements with six degrees of freedom at each node, allowing the simulation of various fixation stabilities by changing the free bending length (offset) of the pins. The model was fixed at the end near the fracture gap of the distal cortical bone fragment in all degrees of freedom. A three dimensional physiological load case (three forces and three moments at that time point during gait of the rats when the axial load component reached its maximum, which was 6 times bodyweight [11]) was applied to the proximal cortical bone fragment using a tent like construct at the end of the proximal cortical bone fragment at the fracture site [21]. The fracture healing process was simulated iteratively over the healing time [18], [19], [21]. At each simulation step, an FE-simulation was performed to calculate the local mechanical stimuli (volumetric strain ε_v_ and distortional strain ε_d_) and subsequently predict the change in the local tissue distribution within the elements of the healing region. Last, Young's modulus (*E*_tiss_) and Poisson’s ratio (*ν*_tiss_) of the FE were updated according to the new tissue composition, *i.e.* the concentration of cartilage (*c_cart_*) and bone (*c_bone_*), using the following rules of mixtures:
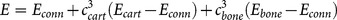
(2)




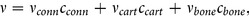
(3)where *E*_conn_, *ν*_conn_, *E*_cart_, *ν*_cart_, and *E*_bone_, *ν*_bone_, are Young’s moduli and Poisson’s ratios of connective tissue, cartilage, and woven bone, respectively.

**Figure 2:**
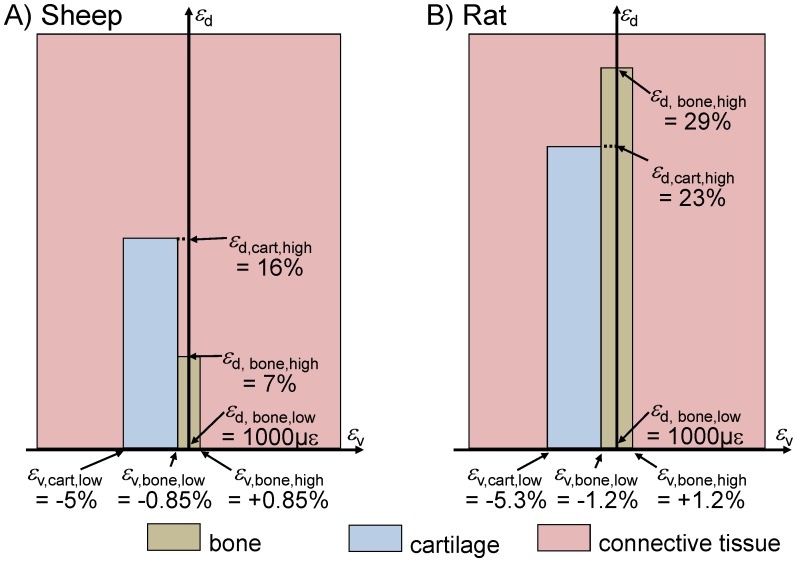
Mechanoregulatory hypothesis for tissue differentiation dependent on the local volumetric (*ε*_v_) and distortional (*ε*_d_) strains. A) Established for sheep, according to Claes and Heigele [9], B) for rat after calibration of the model in the present study.

In contrast to previous simulations of this research group [18], [19], [21], [24], the complexity of the rules for predictions of the tissue differentiation was reduced. Now, bone and cartilage formation were directly linked solely to the mechanical stimuli (ε_v_ & ε_d_). Cartilage formation was allowed for volumetric strains in the range of ε_v,cart,low_ <ε_v_ <ε_v,cart,high_ in combination with distortional strains in the range of ε_d,cart,low_ <ε_d_ <ε_d,cart,high_, whereas bone formation was allowed in the range of the volumetric strains of ε_v,bone,low_ <ε_v_ <ε_v,bone,high_ in combination with distortional strains in the range of ε_d,bone,low_ <ε_d_ <ε_d, bone,high_. Outside these ranges, resorption of these tissues will occur (cf. Fig. 2).

To simulate bone and cartilage formation and resorption, the rate of change in both concentrations was set to 7% per day, since this allowed simulation of a bony callus after 35 days of healing with a size according to previous findings [2].

### Calibration of the underlying mechanoregulatory rules

To adapt the fracture healing algorithm from sheep to rat, the thresholds within the underlying mechanoregulatory tissue differentiation rules (*i.e. ε*_v,cart,low_, ε_v,cart,high_, ε_d,cart,high_, and ε_d,bone,high_, cf. Fig. 2) were varied in 600 different combinations (designs) in a design of experiments (DOE) approach using Latin Hypercube Sampling in the software package optiSLang (Dynardo GmbH, Weimar, Germany). The other parameters defining the ranges of the mechanical stimuli were kept constant to the following values: ε_v,bone,low_ = ε_v,cart,high_, ε_v,bone,high_ = −ε_v,cart,high_, ε_d,cart,low_ = 0, ε_d,bone,low_ = 1000 µ*ε* [25]. The simulations were performed according to the experimental setup of a previously published *in vivo* monitoring study [26]: the fracture healing process was simulated for each design under rigid (K_ax_ = 102 N/mm) and flexible (K_ax_ = 30 N/mm) fixation and the callus stiffness was calculated at each week until 6 weeks (for rigid fixation), or 12 weeks (for flexible fixation). The mean values of differences between simulated and their respective *in vivo* measured callus stiffness were calculated and used to find the best design out of the 600.

### Evaluation and corroboration of the numerical fracture healing model

To evaluate the validity of the calibrated fracture healing algorithm, it was used to simulate 21 *in vivo* scenarios from 9 different experimental rat fracture healing studies from literature (Table 1). Simulation outcomes (*i.e.* simulated callus stiffness) were compared to the *ex vivo* callus stiffness from the respective experiment. Requirements for the included *in vivo* studies were transverse osteotomies of rat femurs, stabilization with external fixators with well described experimental parameters (bodyweight of the animals, fixation stiffness and fracture gap size), and well reported *ex vivo* mechanical testing of the callus stiffness.

**Table 1:** Literature data of different rat experiments which measured the *ex vivo* callus stiffness (*K_c,ex vivo_*) at healing time point *t_H_*. *K_c,sim_* is the callus stiffness, obtained by numerical simulation applying the same bodyweight (*BW*), axial fixation stiffness (*K_ax_*) and fracture gap size (*S_fr_*) as the respective *in vivo* experiment.

Data set #	Reference	*BW* in g	gender (m or f)	*K_ax_* in N/mm	*S_fr_* in mm	*t_H_* in days	*K_c,ex vivo_* in %	*K_c_*_,*sim*_ in %
1	Harrison *et al*. [30]	500	m	46	0.5	63	189±47	115
2	Harrison *et al*. [30]	500	m	46	3	63	n.a.	114
3	Mark *et al*. [5]	400	m	30	2	14	n.a.	2 (2)**
4	Mark *et al*. [5]	400	m	30	2	28	5±1	7 (5)**
5	Mark *et al*. [5]	400	m	30	2	42	50±18	95 (54)**
6	Mark *et al*. [5]	400	m	30	2	84	161±9	114 (117)**
7	Kaspar *et al*. [32]	435	m	34	0.5	56	70–209	113
8	Strube *et al*. [3]	257	f	25	1.5	42	176±38	100
9	Strube *et al* [3]	257	f	10	1.5	42	78±26	108
10	Strube *et al*. [3]	335	f	25	1.5	42	25±9	107
11	Strube *et al*. [3]	335	f	10	1.5	42	38±23	54
12	Strube *et al*. [4]	363	m	10	1.5	42	43−83*	29
13	Strube *et al*. [4]	353	f	10	1.5	42	16−44*	36
14	Claes *et al*. [1]	375	m	74	1	35	53±10	100
15	Claes *et al*. [1]	375	m	10	1	35	35±10	11
16	Mehta *et al*. [37]	366	m	25	1.5	42	52±20	28
17	Mehta *et al*. [31]	326	m	50	1	42	90−132*	99
18	Mehta *et al*. [31]	326	m	50	5	56	0−15*	106
19	Mehta *et al*. [31]	296	f	50	1	42	50−107*	98
*Study used for adjusting the thresholds in the underlying mechanoregulatory rules:*								
20	Recknagel *et al*. [2]	425	m	119	1	35	31−82*	100
21	Recknagel *et al*. [2]	425	m	32	1	35	19−32*	19
*Study used for determining the fracture callus material properties:*								
–	Wehner *et al.* [26]	467	m	102	1	84	126	100
–	Wehner *et al.* [26]	467	m	30	1	84	106	100

*Interquartile range,**simulated with a bodyweight of 450 g.

#1−2: develop a pseudarthrosis model with large fracture gap.

#3–6: examine torsional callus stiffness over healing time.

#7: develops a reproducible standardized rat bone healing model.

#8–11: investigate mechanical impact on fracture healing in aged rats.

#12–13: contribution of mesenchymal stem cells to sex-specific differences in bone healing.

#14–15: investigate the influence of early dynamization on fracture healing.

#16: examine the influence of gender and fixation stability on bone defect healing.

#17–19: develop a reproducible atrophic non-union.

#20–21: investigate how blunt chest trauma impairs fracture healing in rats.

### Prediction of callus stiffness subject to bodyweight, fixation stiffness and fracture gap size

For numerous combinations of different values for bodyweight (*BW)*, fixation stability (*K_ax_*), and fracture gap size (*S_fr_*), fracture healing was simulated with the calibrated and corroborated iterative algorithm described above. For each iteration step, the callus bending stiffness *K*_C_ was calculated by simulating a cantilever bending test. For these time courses of *K*_C_, fit curves with a sigmoidal shape were created using the Curve Fitting Toolbox of Matlab (MathWorks, Ismaning, Germany) with the equation
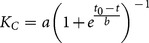
(4)


Using these fit curves, the time to heal (assumed as the time point when the callus stiffness reached 90% of the intact bone stiffness) was calculated for all of these cases and correlated to the initial IFM (defined as the axial internal force in the femoral mid-shaft, which was assumed to be 6 times the bodyweight [11], divided by the axial fixation stiffness) and to the interfragmentary strain (defined as the IFM divided by the fracture gap size). Furthermore, the deviations (minimum *Δ_min_*, median *Δ_median_*, and maximum *Δ_max_*) of the resulting fit curve in comparison to the original simulated curves were calculated to allow evaluation of the fidelity of the fitting function.

## Results

### Determining the material properties of the callus tissues

Using a Young’s modulus of 15,750 MPa for the cortical bone of the intact bone model (Fig. 1) led to a bending stiffness of 182,358 Nmm^2^, which is in good accordance with the *ex vivo* bending stiffness of intact rat femurs (176,805±51,586 Nmm^2^), measured by Recknagel *et al.* [2]. Therefore, the material properties for the cortical shells were incorporated into models 2 and 3 (Fig. 1). Model 2, for the simulation of a callus filled with woven bone with a Young’s modulus of 1,000 MPa, led to a bending stiffness of approx. 100% of the intact bones, typical for a fully bony bridged fracture callus with moderate callus size (callus index = 1.5) [2], [27], [28]. Therefore, the woven bone in model 3 (Fig. 1) was assigned this value for Young’s modulus. Model 3 (Fig. 1) represented a fracture callus with a greater size (callus index = 2) and a fracture gap of 1 mm, filled with connective tissue and cartilage, typical for a healing situation after 35 days under a more flexible fixation [2]. Using a Young’s modulus of 5 MPa for cartilage led to a bending stiffness of 28% to 29% of the intact bones, which is in good agreement with the *ex vivo* results (25±11%) of Recknagel *et al*. [2]. The obtained material properties are summarized in Table 2.

**Table 2:** Material properties obtained for the simulated callus tissues.

Tissue type	Young’s modulus in MPa	Poisson’s ratio
Cortical bone	15,750^a^	0.36^e^
Woven bone	1,000^b^	0.36^e^
Cartilage	5^c^	0.45^e^
Granulation/connective tissue	1^d^	0.4^e^

a Smit *et al*, 2002 [35], obtained as isotropic elastic constant in a poroelastic material model.

b Checa *et al*., 2011 [22], used for the solid phase of immature rat bone in FE study.

c determined via static analysis, cf. S1 Table.

d Leong & Morgan 2008 [36], obtained by nanoindentation on rat fracture callus.

e Simon *et al*., 2011 [18], used in linear elastic FE model approach.

### Calibration of the underlying mechanoregulatory rules

**Figure 3:**
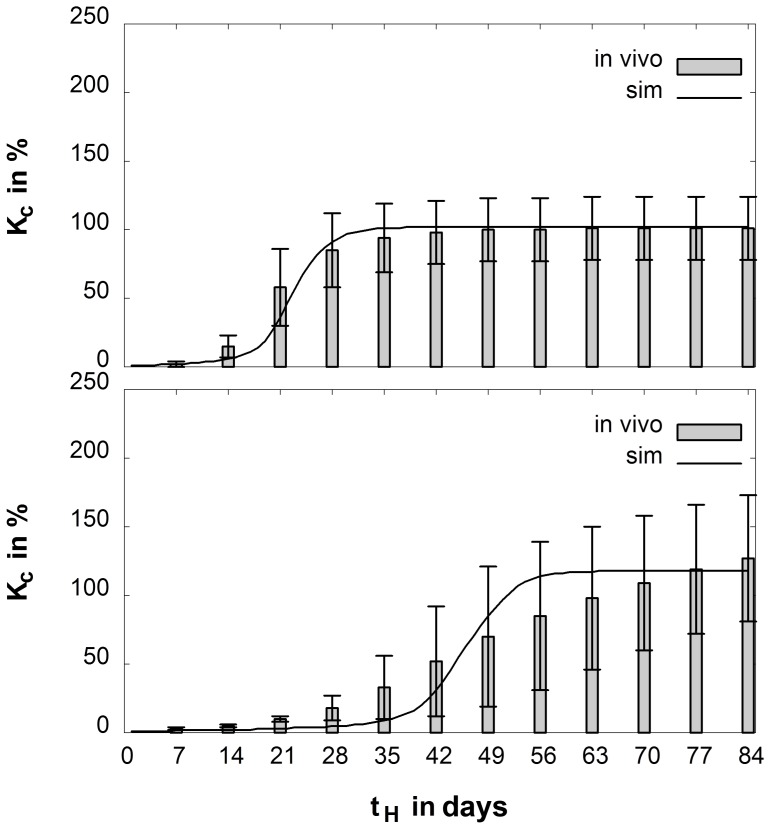
Course of callus stiffness (K_C_) over the healing time (t_H_) under rigid (top) and flexible (bottom) fixation. Bars indicate *in vivo* data (statistical means and 95% confidence intervals) from the rat experiment [29], that was used to calibrate the numerical model, solid line represents the outcome of the numerical simulation after calibration.

**Figure 4:**
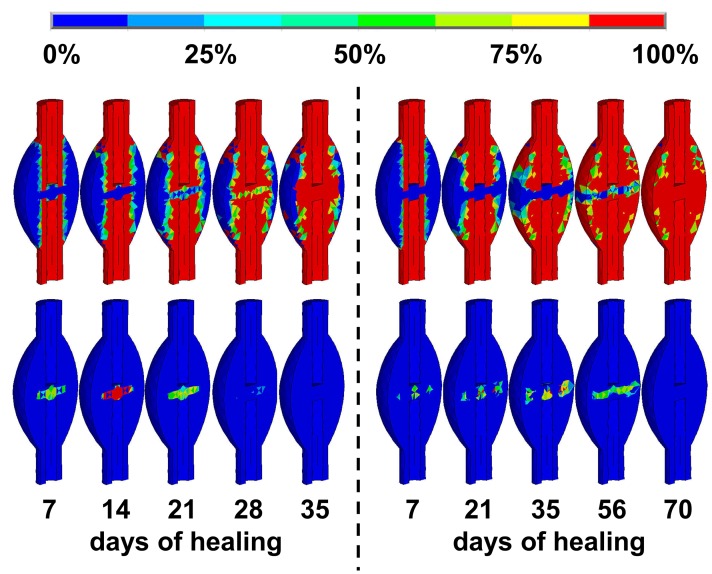
Concentration (tissue type fraction within each finite element) of bone (top) and cartilage (bottom) over the healing time for the rigidly (left) and the flexibly (right) fixated animals.

The best agreement between *in vivo* data and simulations were obtained by shifting the threshold values of the underlying mechanoregulatory rules towards higher levels (ε_v,cart,low_ = −5.3%, ε_v,cart,high_ = −1.2%, ε_d,cart,high_ = 23%, and ε_d,bone,high_ = 29%, cf. Fig. 2B) in comparison to the thresholds that were used in previous simulations (ε_v,cart,low_ = −5%, ε_v,cart,high_ = −0.85%, ε_d,cart,high_ = 16%, and ε_d,bone,high_ = 7%, cf. Fig. 2A) for sheep and humans [18], [19], [21]. Applying these calibrated thresholds, the time course of the callus stiffness was predicted in good accordance with the *in vivo* data from the previous study [29] (Fig. 3). Regarding the tissue distribution after 35 days of healing, the simulations yield a bridged, bony callus of moderate size under rigid fixation (callus index approximately 1.5), and a larger bony callus (callus index approximately 2.0) under flexible fixation, with a remaining fracture gap that contains connective tissue and cartilage (Fig. 4). These predictions correspond well to respective, previous findings [2], [29].

### Evaluation and corroboration of the numerical fracture healing model

**Figure 5:**
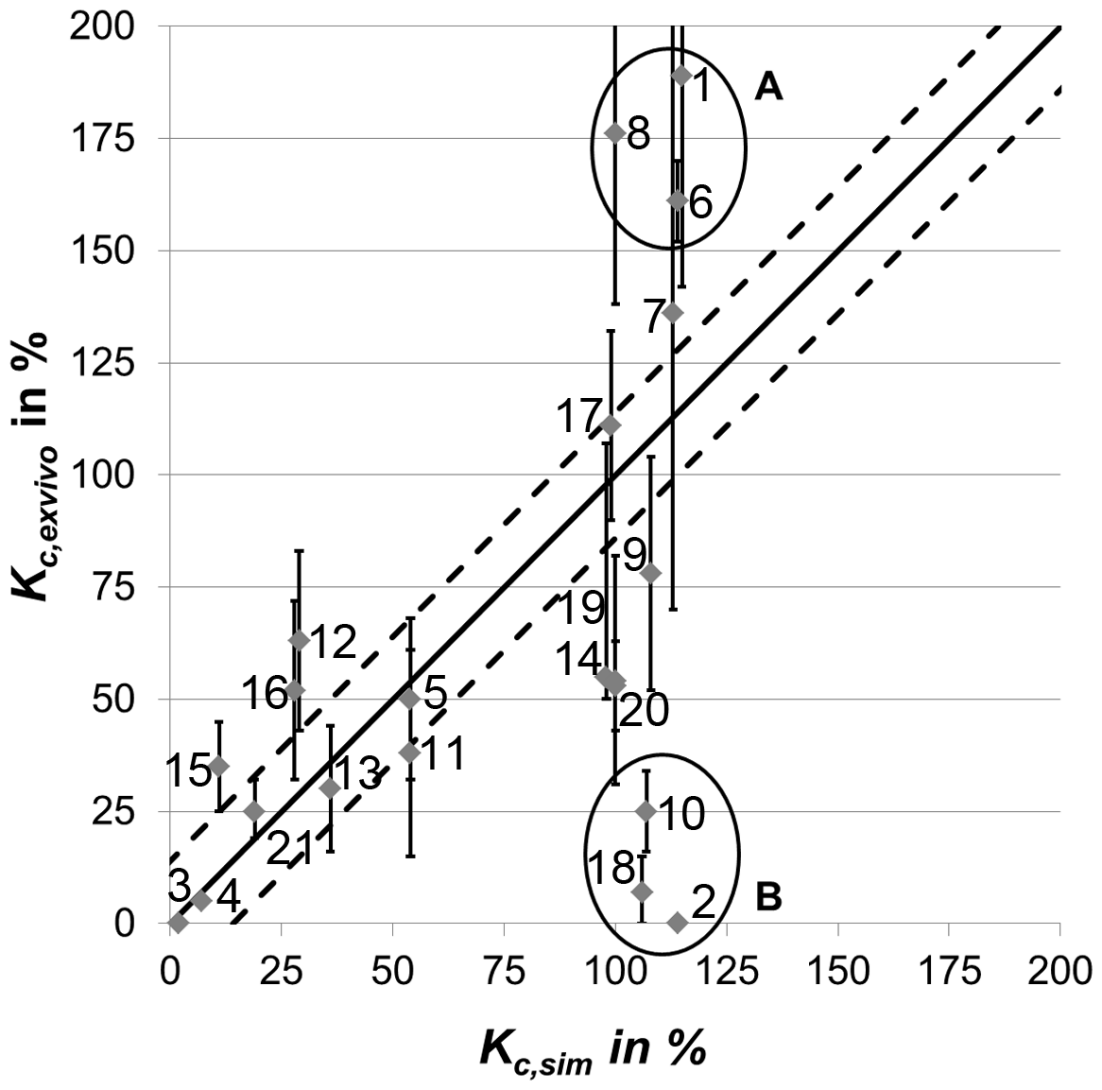
Comparison of simulated (*K_c,sim_*) and *ex vivo* measured (*K_c,exvivo_*) callus stiffness of experimental studies from literature. The points and error bars indicate mean and standard deviations or median and interquartile ranges. Numbers of the single *in vivo* data points refer to Table 1. The solid line represents perfect agreement and the dashed lines indicate uncertainty ranges of the simulations due to variations in gap size, free bending length of the pins, and bodyweight of the rats (±14% of the intact bone stiffness, determined in a preceded sensitivity analysis as described in the discussion section). In some cases, numerical predictions clearly underestimated the callus stiffness after bony bridging (group A) and in some cases, successful healing with high callus stiffness was predicted although no healing was observed *in vivo* and low callus stiffness was measured *ex vivo* (group B).

The simulations of *in vivo* rat studies from literature showed good agreement for several combinations of fixation stiffness and fracture gap size qualitatively (successful healing or not) and quantitatively (direct comparison of the simulated and the *ex vivo* measured callus stiffness at a specific time point – Fig. 5). However, some large differences exist (Table 1). First, at large fracture gap sizes (3 mm & 5 mm), a successful healing process with high callus stiffness was predicted, whereas there was no measureable callus stiffness observed *in vivo* [30], [31]. Second, a successful healing was simulated for old female rats after 42 days with a predicted callus stiffness of 107%, whereas the *ex vivo* measured callus stiffness was still low at 25±9% [3]. Furthermore, the simulated stiffness of fracture calluses, which are completely filled with woven bone, were in some cases lower [3], [5], [30] (differences up to 76% of intact bone stiffness) or higher [1] (differences up to 47% of intact bone stiffness) than the measured *in vivo* results (Table 1).

### Prediction of callus stiffness subject to bodyweight, fixation stiffness and fracture gap size

**Figure 6:**
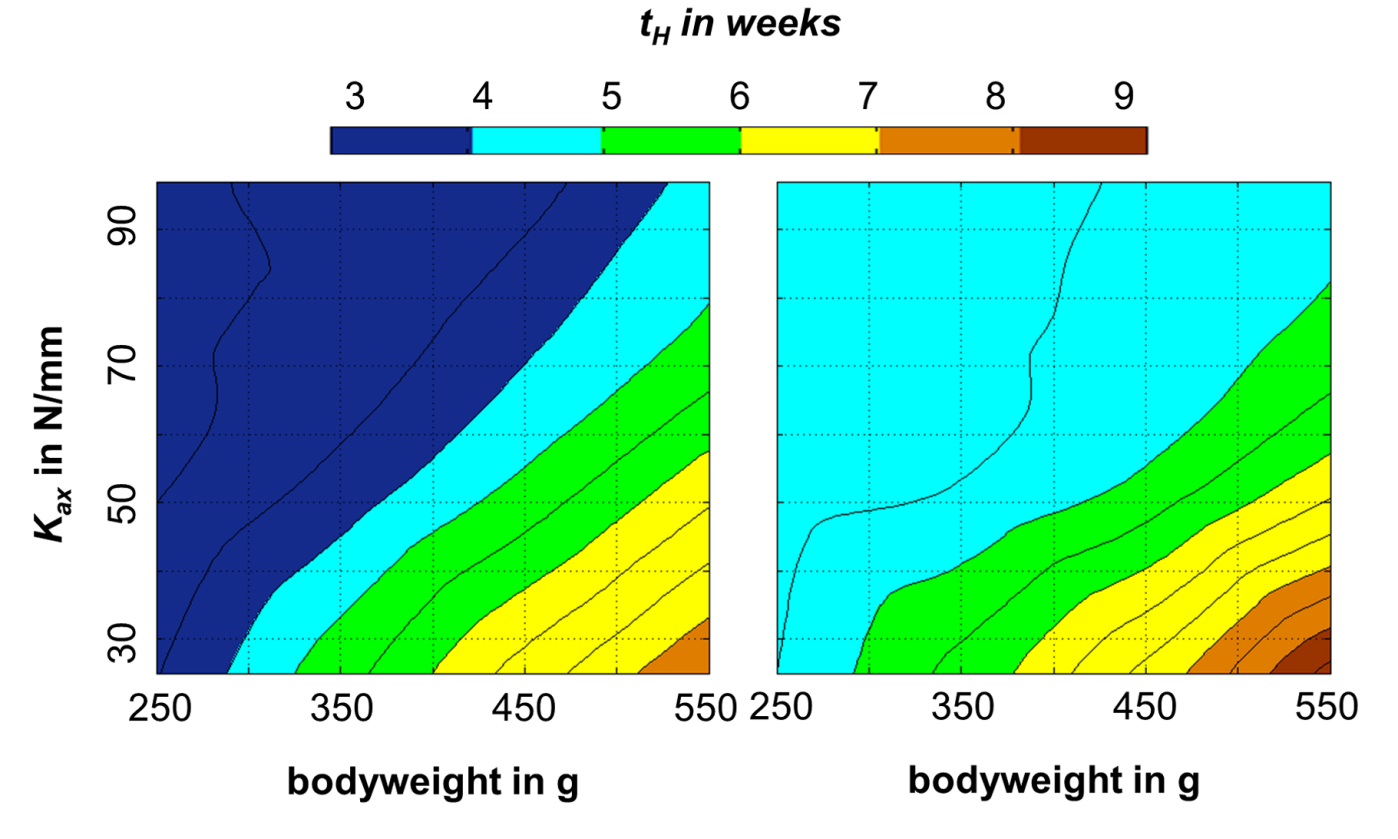
Time to heal (*t_H_*, defined as time point, when callus stiffness reached 90% of intact bone stiffness) in relation to bodyweight of the rats and axial stiffness (*K_ax_*) of the external fixator at a fracture gap size of 1 mm (left) and 2 mm (right), calculated using the parameters given in supplemental material (cf. S2 Table).

Using fit curves with a sigmoidal shape, the simulated course of callus stiffness over the healing time could be replicated well with a maximal error of <5% of the intact bone stiffness (cf. S2 Table, which provides fit curve parameters for numerous different combinations of bodyweight, fixation stiffness and fracture gap size). The time to heal (assumed to be the time point when the callus stiffness reached 90% of the intact bone stiffness) was prolonged for larger gap sizes, lower fixation stiffness and higher bodyweights of the rats (Fig. 6).

## Discussion

In this study, a simplified version of an already existing fracture healing algorithm was successfully adapted to the healing process in rats by calibrating the thresholds of the underlying mechanoregulatory tissue differentiation rules. To simulate the time course of the callus stiffness and the patterns of bone and cartilage formation according to *in vivo* findings in rats [2], [29], the threshold values of the mechanical stimuli (volumetric and distortional strains) had to be shifted towards higher load levels, which is in contrast to the simulations of Checa and colleagues [22]. In their study, the mechanical conditions to allow bone formation had to be shifted towards higher load levels for the sheep. However, the authors used a different numerical model for simulating the healing processes and used a different *in vivo* dataset for evaluating the simulations. Due to the differences of both numerical models (Checa *et al*. used a poroelastic description of the material properties, and a linear combination of the fluid flow and the distortional strain as mechanical stimulus and simulated several cell activities such as migration and proliferation), it is hard to directly compare their simulation results with the findings of this study. Therefore, it remains unclear if the differences in the necessary adaptation of the load levels from the sheep to the rat are due to differences between either the numerical models or the *in vivo* data used for evaluation. Further simulations with both models using the same *in vivo* data are required to clarify this issue.

In the present study, several published *in vivo* studies using rats were simulated to test the validity of the applied algorithm for other combinations of the experimental parameters bodyweight, fixation stiffness and fracture gap size (Table 1). With the exception of healing scenarios in which the healing process was seemingly more influenced by biological factors (large fracture gaps [30], [31] and age [3]), the achieved numerical predictions agreed particularly well with the respective *in vivo* findings (Table 1). Taking the variations of the *ex vivo* measured callus stiffness into account, which showed standard deviations of up to 47% [30] and interquartile ranges of 139% [32] of the intact bone stiffness, there were only 6 of 21 cases showing a considerable mismatch between simulations and *in vivo* data.

A preceding sensitivity study using the calibrated numerical model showed that varying the gap size by 0.1 mm, the offset of the fixator body by 0.5 mm, and the bodyweight by 25 g (all standard deviations) led to standard deviations of up to 14% for the rigid group and up to 20% for the flexible group and a maximum range of the callus stiffness of up to 57% (rigid) and up to 77% (flexible) of the intact bone stiffness (S1 Fig.). Aside from biological differences between the rats, which also have an influence on the healing process, these simulation results explain variations of the *ex vivo* measured callus stiffness due to variations of experimental parameters. For example, the range of the bodyweight of the rats used in the study of Mark *et al*. [5] was 350 to 450 g. Simulating the healing process with a bodyweight of 400 g led to a difference of 45% of the intact bone stiffness at the 6 weeks healing time point (50±18% [5] vs. 95%) whereas this difference was minimal (50±18% [5] vs. 52%), when the healing process was simulated with 450 g bodyweight (Table 1) indicating a good agreement of the achieved numerical predictions considering variations in the experimental parameters.

However, there were several necessary assumptions and simplifications that were made. Firstly, material behavior was simplified to linear elastic, isotropic behavior. In particular, this limits the model outcomes in the first phases of healing when tissues are still very flexible and show a distinct fluid phase. Secondly, the adjusted rules for tissue differentiation are simplified to a minimum, compared to previous numerical simulation studies of this group [18], [19], [33], [34] and completely focus on mechanoregulation without considering any biological influences. Thirdly, as previously mentioned, the predictions for healing processes accompanied by large fracture gaps (3 & 5 mm) did not fit well to the *in vivo* results (Table 1). Enlarging the fracture gap in the applied numerical model leads to a delayed healing since it takes longer to bridge the fracture gap with the same rate of bone formation (S2 Table). However, as long as this bone formation rate will not be set to zero and the IFM will be high enough to prevent a mechanical understimulation of the callus tissue, the model will predict successful healing for all gap sizes. This suggested that the causality of a critical size defect cannot be explained by mechanical stimuli solely and that biological factors must be considered. To include the simulation of non-unions in this model as well, the bone formation rate could be described as a function of the healing time. Thereby a decay of the biological factors promoting tissue development over the healing time could be incorporated allowing the simulation of non-unions as well. Since the exact, underlying biological mechanisms are, so far, not known to predict the tissue development in those cases, the present simulations were focused on the influence of the biomechanical factors on the healing process.

In conclusion, the present numerical model was considered valid for normal healing processes of transverse osteotomies in rats with fracture gap sizes in the range of 0.5 to 2 mm and bodyweights in the range of 250 to 550 g, stabilized by external fixators with an axial fixation stiffness in the range of 10 to 119 N/mm. The data presented in this study might help to improve new investigations of fracture healing in rats by predicting the time course of the healing process prior to the animal studies and allowing the transfer of new *in vivo* findings to previous studies with different bodyweight, fixation stiffness and fracture gap sizes.

## Supporting Information

S1 Fig **Course of callus stiffness (KC) over the healing time (tH) under rigid and flexible fixation.**
(PDF)

S1 Table **Bending stiffness calculated with the three finite element models in relation to the Young’s moduli of the different tissue types.**
(PDF)

S2 Table **Parameters of the sigmoid function (** ***a, b, t0*** **- cf.** **equation 4** **), used to predict the time course of the callus stiffness (** ***Kc*** **) in percent (%) of the intact bones under different combinations of** ***BW*** **,** ***Kax*** **, and** ***Sfr*** **.**
(PDF)
