# Supplementary material for: Prediction of the Time Course of Callus Stiffness as a Function of Mechanical Parameters in Experimental Rat Fracture Healing Studies - A Numerical Study
Source: PLoS One. 2014 Dec 22;9(12):e115695. doi: 10.1371/journal.pone.0115695 (PMC4274111; doi:10.1371/journal.pone.0115695)
Supplement: S1 Fig — Course of callus stiffness (KC) over the healing time (tH) under rigid and flexible fixation. (PDF) [file pone.0115695.s001.pdf]

Supplementary material – Figure S1

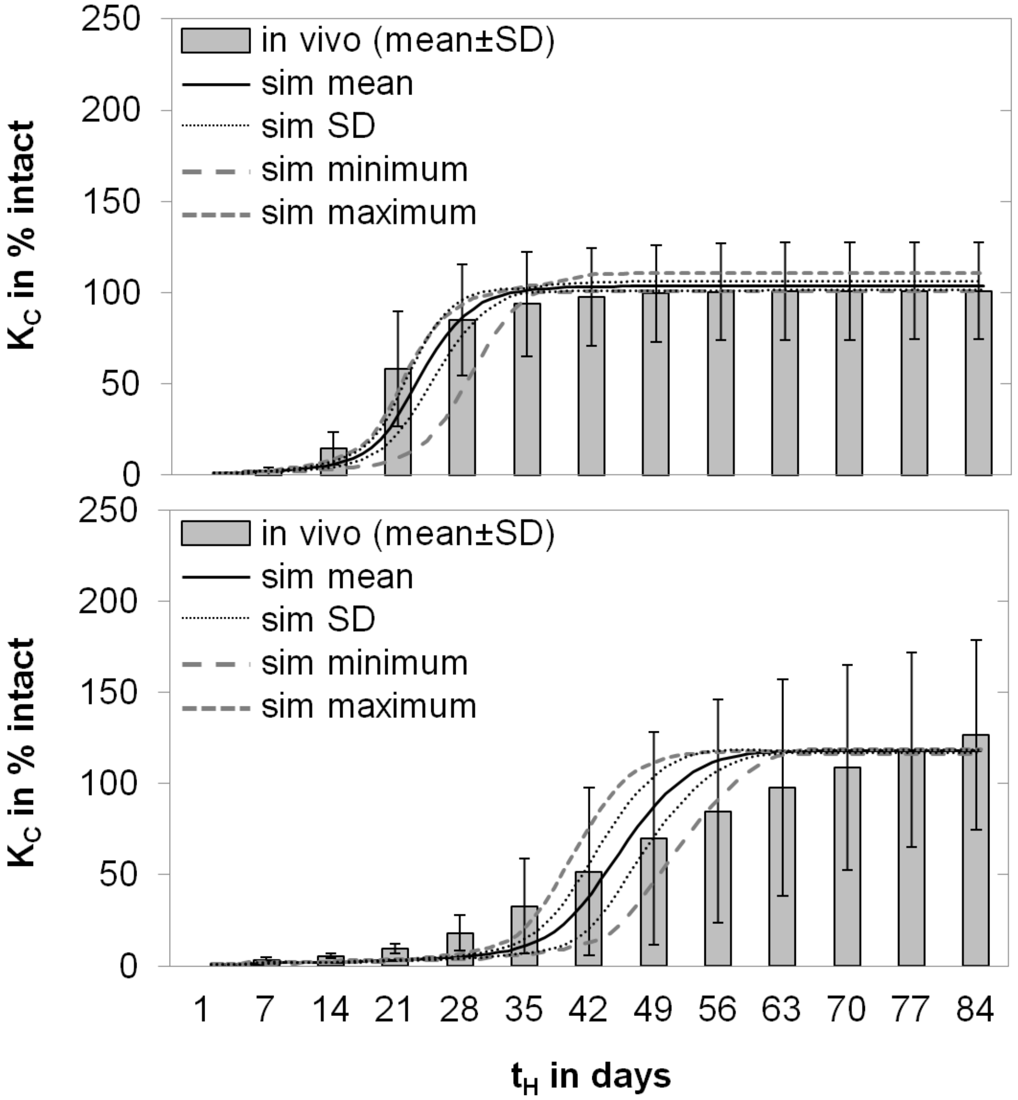

Figure S1: Course of callus stiffness (K_C) over the healing time (t_H) under rigid (top) and flexible (bottom) fixation. Bars indicate *in vivo* data (statistical means and 95% confidence intervals) from the rat experiment [26]. Lines show results of a sensitivity study using the calibrated numerical model. Therefore, the following parameters were varied as follows to create 30 different combinations: gap size by 0.1 mm, offset of the fixator body by 0.5 mm, and the bodyweight by 25 g (all standard deviations). Progresses of statistical mean, standard deviation, minimum and maximum data are shown. Standard deviations were up to 14% for the rigid and up to 20% for the flexible group. Maximum range of the callus stiffness was up to 57% (rigid) and 77% (flexible) of the intact bone stiffness.
