# Supplementary material for: Prediction of the Time Course of Callus Stiffness as a Function of Mechanical Parameters in Experimental Rat Fracture Healing Studies - A Numerical Study
Source: PLoS One. 2014 Dec 22;9(12):e115695. doi: 10.1371/journal.pone.0115695 (PMC4274111; doi:10.1371/journal.pone.0115695)
Supplement: S1 Table — Bending stiffness calculated with the three finite element models in relation to the Young’s moduli of the different tissue types. (PDF) [file pone.0115695.s002.pdf]

Supplementary material – Table S1

Table S1: Bending stiffness was calculated with the three finite element models (Figure 1) in relation to the Young's moduli of the different tissue types. Asterisks (*) mark the best agreement with literature data. The respective material parameters were assigned to the subsequent model.

FE model	E_{cort} in MPa	E_{bone} in MPa	E_{endost} in MPa	E_{gap} in MPa	EI_{FE} in Nmm²	EI_{FE} in % of intact
1	15,750[a]	-	-	-	182,358*	100*
2	15,750[a]	200[b]	-	-	102,004	56
2	15,750[a]	1,000[c]	-	-	176,342	97*
3	15,750[a]	1,000[c]	1	3[b]	34,352	19
3	15,750[a]	1,000[c]	3[b]	3[b]	35,360	19
3	15,750[a]	1,000[c]	27[b]	3[b]	40,130	22
3	15,750[a]	1,000[c]	200[b]	3[b]	53,447	29
3	15,750[a]	1,000[c]	1	5	50,602	28*
3	15,750[a]	1,000[c]	5	5	52,333	29
3	15,750[a]	1,000[c]	27[b]	5	56,620	31
3	15,750[a]	1,000[c]	200[b]	5	69,332	38
3	15,750[a]	1,000[c]	1	10[c]	79,165	43
3	15,750[a]	1,000[c]	10[c]	10[c]	81,842	45
3	15,750[a]	1,000[c]	27[b]	10[c]	84,602	46
3	15,750[a]	1,000[c]	200[b]	10[c]	95,807	53

[a]Smit *et al.*, 2002 [34]; [b]Leong & Morgan, 2008 [35]; [c]Checa *et al.*, 2011 [22]
