# Supplementary material for: Prediction of the Time Course of Callus Stiffness as a Function of Mechanical Parameters in Experimental Rat Fracture Healing Studies - A Numerical Study
Source: PLoS One. 2014 Dec 22;9(12):e115695. doi: 10.1371/journal.pone.0115695 (PMC4274111; doi:10.1371/journal.pone.0115695)
Supplement: S2 Table — Parameters of the sigmoid function ( a, b, t0 - cf. equation 4 ), used to predict the time course of the callus stiffness ( Kc ) in percent (%) of the intact bones under different combinations of BW , Kax , and Sfr . (PDF) [file pone.0115695.s003.pdf]

Supplementary material – Table S2

Table S2: Parameters of the sigmoid function (***a, b, t_0*** - cf. equation 4) were used to predict the time course of the callus stiffness (***K_c***) in percent (%) of the intact bones under different combinations of ***BW***, ***K_{ax}***, and ***S_{fr}*** along with the deviations (***Δ_{min}***, ***Δ_{median}***, and ***Δ_{max}***) of the resulting curve in comparison to the original simulations.

BW in g	***K_{ax}*** in N/mm	***S_{fr}*** in mm	***a*** in %	***b***	***t_0*** in days	***Δ_{min}*** in %	***Δ_{median}*** in %	***Δ_{max}*** in %
250	25	0.5	101.1	2.716	20.07	-2.4	0.1	1.5
250	25	1.0	100.0	2.437	22.42	-2.1	0.0	1.0
250	25	1.5	101.0	2.295	23.80	-2.6	0.0	1.8
250	25	2.0	99.8	2.307	26.38	-2.5	-0.2	2.2
250	52	0.5	100.0	2.386	18.08	-2.4	0.0	1.6
250	52	1.0	96.9	2.135	18.66	-2.4	-0.1	1.6
250	52	1.5	98.0	2.133	20.96	-2.4	0.0	1.6
250	52	2.0	96.6	2.140	24.41	-2.8	-0.5	1.9
250	74	0.5	99.0	2.353	16.87	-2.0	0.0	1.6
250	74	1.0	96.0	2.113	18.27	-2.3	0.0	1.4
250	74	1.5	97.6	2.033	20.48	-2.1	-0.4	1.3
250	74	2.0	93.9	2.074	22.62	-2.6	-0.1	1.7
250	97	0.5	98.1	2.244	15.82	-2.2	0.0	1.7
250	97	1.0	95.8	2.050	17.99	-2.3	-0.2	1.3
250	97	1.5	96.0	1.992	19.47	-2.2	0.0	1.5
250	97	2.0	91.9	2.112	20.48	-2.0	-0.1	1.7
350	25	0.5	107.2	3.289	24.82	-2.5	0.1	0.9
350	25	1.0	113.3	3.171	32.87	-3.0	0.0	1.1
350	25	1.5	113.1	2.666	33.93	-3.4	-0.1	2.3
350	25	2.0	113.1	2.723	35.95	-3.6	-0.1	1.6
350	52	0.5	104.1	3.048	22.25	-2.5	0.1	0.9
350	52	1.0	103.0	2.555	24.58	-2.6	0.0	1.5
350	52	1.5	101.9	2.442	23.25	-2.0	-0.1	1.3
350	52	2.0	102.9	2.549	26.84	-2.6	-0.1	1.9
350	74	0.5	102.1	2.835	20.52	-2.4	0.1	1.2
350	74	1.0	99.0	2.331	20.36	-2.3	-0.1	1.9
350	74	1.5	100.0	2.323	22.15	-2.5	0.0	1.5
350	74	2.0	101.6	2.507	25.65	-2.6	-0.4	2.2
350	97	0.5	101.1	2.625	19.10	-2.3	0.1	1.7

350	97	1.0	98.8	2.441	20.09	-2.7	-0.2	1.7
350	97	1.5	99.9	2.279	21.68	-2.6	-0.1	1.6
350	97	2.0	98.8	2.361	24.36	-2.2	-0.2	2.6
450	25	0.5	116.6	3.914	31.90	-3.1	0.0	2.0
450	25	1.0	117.6	3.368	43.00	-4.0	-1.0	2.2
450	25	1.5	117.6	3.151	44.92	-4.4	-1.0	2.4
450	25	2.0	116.6	3.166	46.05	-3.6	-1.0	2.2
450	52	0.5	110.0	3.549	25.58	-2.4	0.0	0.9
450	52	1.0	113.3	3.121	31.83	-3.0	0.1	1.7
450	52	1.5	112.0	2.574	31.82	-3.9	0.0	3.1
450	52	2.0	112.1	2.888	32.11	-2.8	0.1	1.8
450	74	0.5	108.1	3.394	24.06	-2.3	0.1	1.1
450	74	1.0	106.7	2.783	26.10	-2.5	-0.3	2.4
450	74	1.5	104.7	2.638	24.76	-2.5	-0.3	2.2
450	74	2.0	107.6	2.854	28.08	-2.8	-0.4	2.3
450	97	0.5	105.1	3.158	22.30	-2.4	0.1	1.5
450	97	1.0	100.9	2.464	21.71	-2.7	-0.1	2.2
450	97	1.5	103.8	2.603	24.12	-3.0	-0.2	2.1
450	97	2.0	105.9	2.797	27.11	-2.6	-0.1	1.9
550	25	0.5	119.7	4.017	38.31	-4.0	-0.7	2.0
550	25	1.0	119.3	4.105	51.36	-4.5	-1.1	3.4
550	25	1.5	119.5	4.074	55.37	-4.0	-1.0	2.5
550	25	2.0	119.2	4.053	59.59	-4.1	-1.0	2.0
550	52	0.5	117.6	3.887	32.45	-3.7	0.1	1.4
550	52	1.0	117.5	3.209	40.61	-4.3	-0.7	2.8
550	52	1.5	116.5	3.043	41.67	-4.7	-1.0	3.7
550	52	2.0	115.6	3.233	40.58	-3.7	-0.9	2.6
550	74	0.5	113.3	3.664	28.15	-3.0	0.2	1.3
550	74	1.0	114.4	3.297	31.77	-2.9	0.1	1.5
550	74	1.5	113.1	2.942	31.50	-3.4	0.0	1.9
550	74	2.0	113.2	2.915	32.43	-3.9	0.1	2.3
550	97	0.5	111.2	3.639	25.72	-2.5	0.1	1.4
550	97	1.0	110.1	3.083	28.20	-2.5	0.0	1.8
550	97	1.5	108.0	2.812	26.86	-3.4	0.0	2.8
550	97	2.0	109.9	3.031	29.08	-3.0	-0.1	2.2
